# Post-Viral Longitudinally Extensive Transverse Myelitis: A Case Report

**DOI:** 10.7759/cureus.62033

**Published:** 2024-06-09

**Authors:** Sangram Mangudkar, Sindhuri Goud Nimmala, Mahabir P Mishra, Vineetha N Giduturi

**Affiliations:** 1 Internal Medicine, Dr. D. Y. Patil Medical College, Hospital and Research Centre, Pune, IND

**Keywords:** intravenous immunoglobulins (ivig), neurotropic virus, infectious, dengue fever, longitudinally extending transverse myelitis

## Abstract

Longitudinally extensive transverse myelitis is a rare neurological manifestation caused by dengue infection. Here, we describe the uncommon presentation of a 24-year-old male with fever and maculopapular rash followed by flaccid quadriparesis with acute urinary retention. Magnetic resonance imaging of the whole spine with contrast revealed a long-segment ill-defined hyperintense signal noted in the cord. The patient was managed conservatively with intravenous steroids and later intravenous immunoglobulins. The patient is on regular follow-up and doing well. Currently, the patient is on tablet prednisolone with a tapering dose.

## Introduction

Longitudinally extensive transverse myelitis refers to a pattern of extensive spinal cord injury, that clinically manifests as unilateral or bilateral weakness, bilateral sensory symptoms, and bladder and/or bowel dysfunction. Longitudinally extensive transverse myelitis is usually associated with neuromyelitis optica, autoinflammatory disorders, other inflammatory disorders, and infections. Infections resulting in longitudinally extensive transverse myelitis are not frequently seen [1]. Dengue virus presenting with longitudinally extensive transverse myelitis is a rare occurrence. The most frequent arboviral infection affecting people all over the world is dengue fever, which is sometimes referred to as break-bone fever. The disease is often endemic, but there have been multiple epidemics documented. Neurological complications associated with the dengue virus are reported in 0.5% to 6% of dengue fever cases [2]. According to Tomar et al., spinal cord involvement in dengue virus occurs mostly in the form of transverse myelitis [2]. Around six adult cases have been reported in India [3]. Our patient's presenting symptom was para-infectious transverse myelitis, an extremely uncommon manifestation.

## Case presentation

A 24-year-old male inhabitant of Pune, India experienced fever and maculopapular rash for four days. He experienced tingling and numbness in his upper and lower limbs, along with a band-like sensation just below the nipple region for two days. He additionally complained of weakness in his lower limbs and urinary retention. However, he did not experience any bowel symptoms. A sudden onset of high-grade fever associated with chills and rigor was noticed by the patient with an extensive maculopapular, blanching rash all over the body. Following that, tingling and numbness occurred in both the upper and lower limbs, in addition to weakness in the form that he was unable to walk and had difficulty standing up from a squatting position and also had trouble lifting both arms above his head. His symmetrical weakness progressed from distal to proximal in all four limbs over a span of two days. The patient did not suffer from any breathlessness, chest pain, cough, sore throat, difficulty swallowing, difficulties with speech, or had recently received any vaccination. He had no significant medical history, including back trauma or fall. His temperature was 101 degrees Fahrenheit. There were no signs of pallor, icterus, cyanosis, clubbing lymphadenopathy, or edema. The patient’s pulse rate was 96 beats per minute, and a blood pressure of 118/80 mmHg was not associated with postural hypotension. His respiratory rate was 18 cycles per minute, oxygen saturation was 98% on room air, and his Glasgow Coma Scale score was 15/15. His single breath count was 28 per minute.

The patient’s neurological examination showed ascending symmetrical weakness and he was able to move his limbs against gravity on both sides at the shoulder, the elbow, and the wrist. Power was grade of 2/5 at the hip, knee, and ankle joints suggesting movement is possible when gravity is excluded. The conjunctival and corneal reflexes were normal bilaterally. The abdominal reflex was absent at all levels, bilaterally. The Babinski reflexes were mute bilaterally. Finally, the deep tendon reflexes were depressed bilaterally. The patient’s cranial nerve examination was within the normal. The pain sensation and the crude and fine touch decreased from the epigastric region up to the knee joint. The vibration and joint position sensations were normal. There were no involuntary movements. Relevant laboratory investigations have been mentioned below in Table 1

**Table 1:** Laboratory investigations with normal range mentioned correspondingly.

Investigation	Value	Range
Hemoglobin	15.6 gm/dl	11.5-15 gm/dl
Total Leucocyte count	4800/microlt	4000-10000/microlt
Platelet count	107000/microlt	150000-410000/microlt
Creatinine	0.85 mg/dl	0.6-1.2 mg/dl
Dengue	NS1 Negative	Negative <0.90 units Equivocal 0.9 to <1.1 units Positive >/= 1.1 units
Dengue	IgM Positive (five-fold rise)	Negative <0.90 units Equivocal 0.9 to <1.1 units Positive >/= 1.1 units

The serum electrolyte levels are within normal limits. Human immunodeficiency viruses, hepatitis B surface antigen, hepatitis C virus, the venereal disease research laboratory test, and serology were negative. Rapid malaria was negative. Qualitative dengue reverse transcriptase polymerase chain reaction analysis was positive.

CSF analysis revealed CSF proteins of 54 mg/dl wherein there was mild elevation (15-45 mg/dl is the normal range). CSF glucose was reported to be 54 mg/dl (40-80 mg/dl is the normal range) for a corresponding blood glucose of 102. The total leucocyte count in CSF analysis was 70/cumm out of which neutrophils accounted for 10% and the rest 90% were lymphocytes. CSF adenosine deaminase was 1.31 U/L(0-5 U/L is the normal range). CSF culture was negative for bacteria and fungus.

Serum neuromyelitis optica myelin oligodendrocyte glycoprotein, serum antinuclear antibody tests, anti-neutrophil cytoplasmic antibodies profile, and CSF for oligoclonal bands, all were negative.

**Figure 1:**
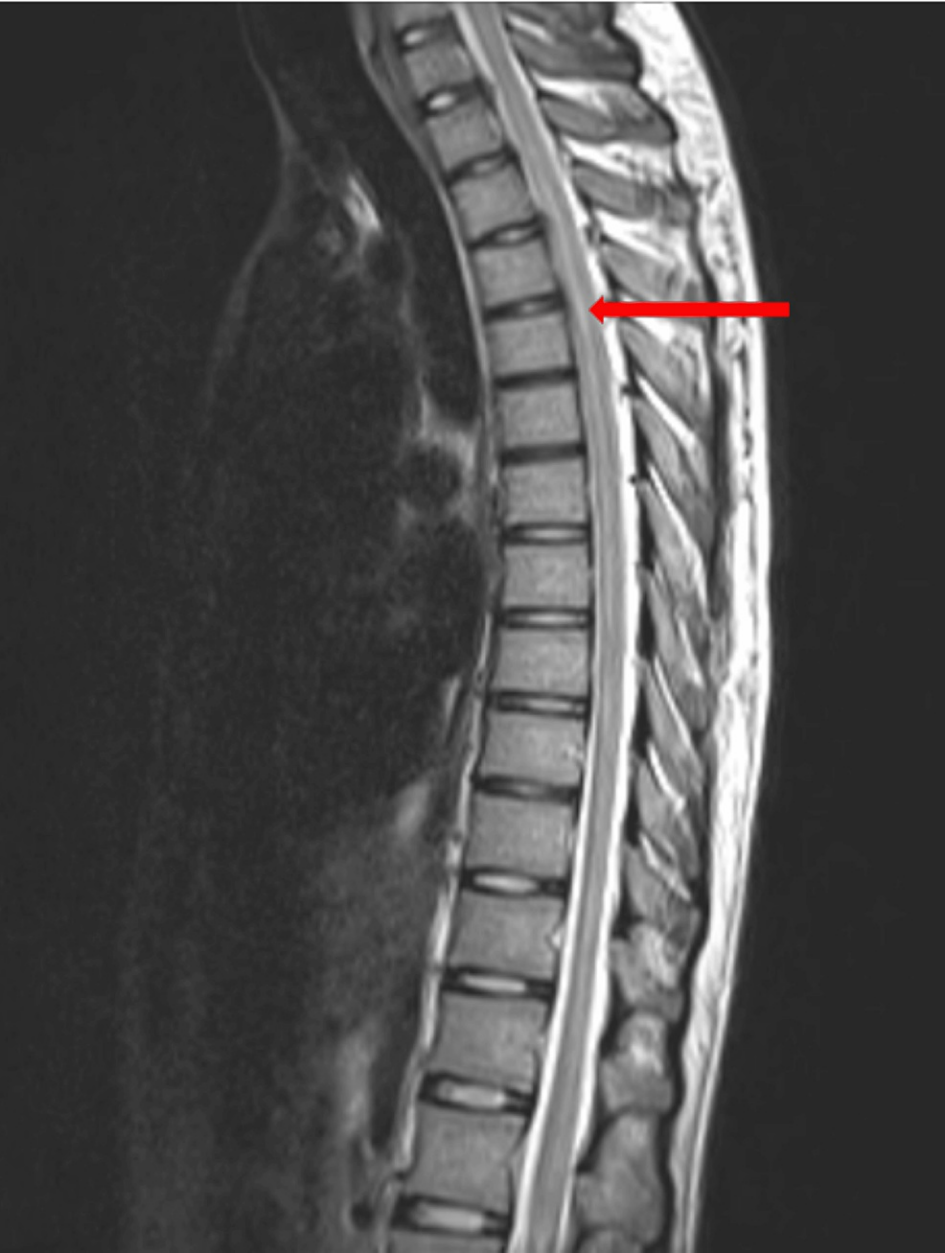
MRI whole spine with the dedicated cervical and lumbar region with contrast: T2/STIR long-segment ill-defined hyperintense signal noted in the cord extending from C2 vertebral body to upper half of L2 vertebral body with the possibility of transverse myelitis (red arrow).

**Figure 2:**
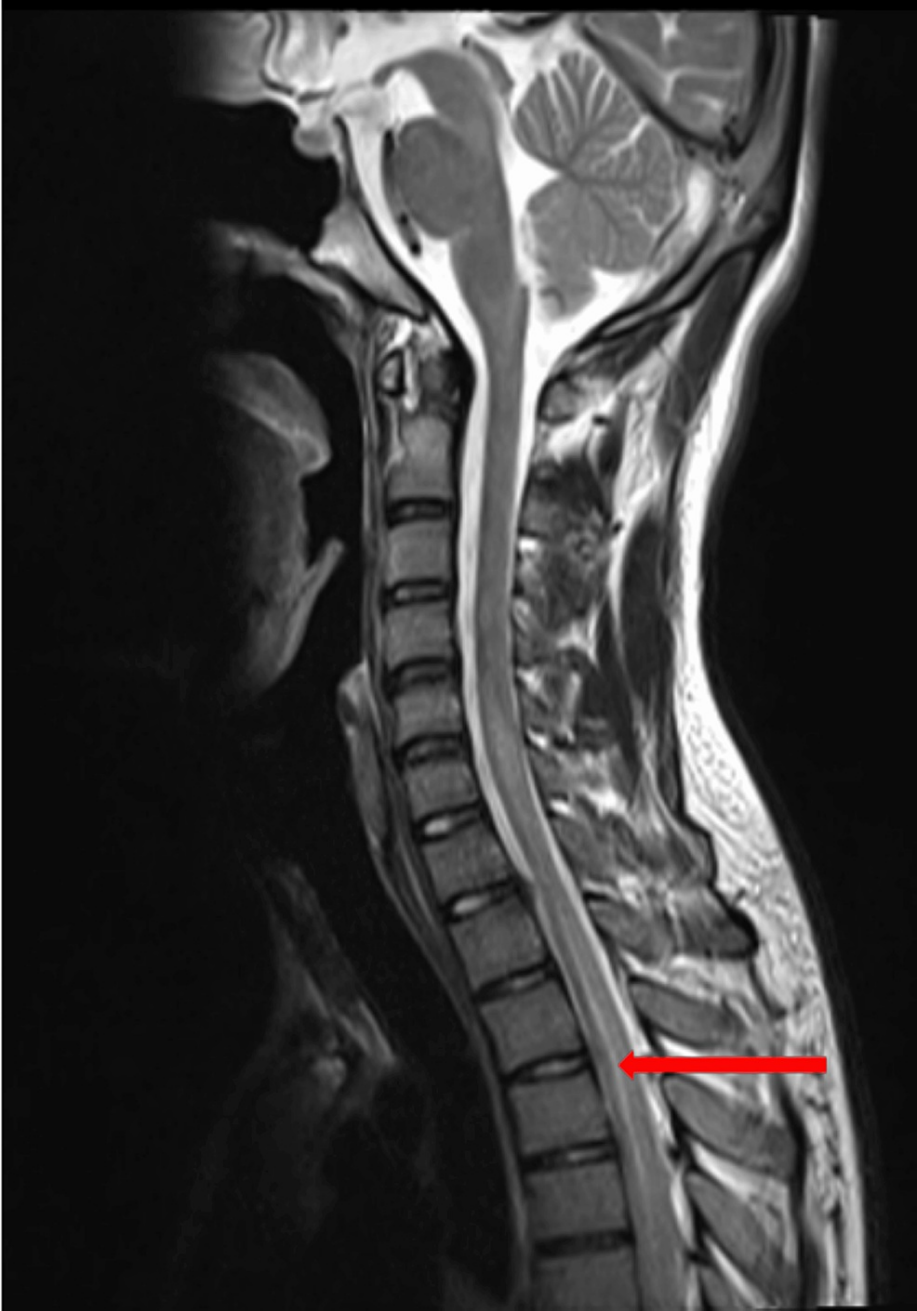
MRI whole spine with the dedicated cervical and lumbar region with contrast: T2/STIR long-segment ill-defined hyperintense signal noted in the cord extending from C2 vertebral body to upper half of L2 vertebral body with the possibility of transverse myelitis (red arrow).

There were no neuroparenchymal abnormalities detected during the MRI brain plain plus contrast. An MRI of the whole spine with dedicated cervical and dorso-lumbar spine was performed with contrast. The images revealed a long segment with an ill-defined hyperintense signal in the spinal cord from the second cervical vertebra to the upper half of the second lumbar vertebral body, suggestive of longitudinally extensive transverse myelitis as represented in Figure 1 and Figure 2.

Fundus examination was within normal limits, with no evidence of papilledema. On 2D echocardiography, a left ventricular ejection fraction of 60%, with no regional wall motion abnormality was found.

The patient was catheterized in view of urinary retention. Steroid pulsing with an IV injection of 1 gram of methylprednisolone in 100 ml of normal saline (NS) once a day was administered for five days, followed by a daily 40 mg tablet of prednisolone, which was gradually reduced every seven days. Supportive symptomatic management has been given to the patient. Intravenous fluids, oral rehydration therapy, and antipyretics were given as supportive therapy. The patient also underwent bladder rehabilitation and training. His lower limb strength increased on day 3 and he was able to perform active movement against gravity (3/5) while his power was normal (5/5) by day 9. However, the patient did not perceive bladder sensation. After two days, the patient was administered 2 grams per kilogram of intravenous immunoglobulins over a period of five days (from day 10 to day 15), along with a 0.4 ml injection of enoxaparin throughout the same five-day period. The patient was discharged on day 16 with a Foley catheter in situ and is currently on frequent follow-up care. One month post-discharge, his muscle strength in all four limbs had increased to grade 5, and was able to walk unaided. The patient exhibited exaggerated deep tendon reflexes. His bladder dysfunction has also improved, and he is currently not catheterized.

## Discussion

The dengue virus belongs to the Flavivirus genus under the *Flaviviridae* family and possesses single-stranded RNA. It is spread in the tropical and subtropical regions of India by *Aedes aegypti*. The incubation phase lasts four to six days. There are four serotypes of dengue virus that are antigenically related: DENV1, DENV2, DENV3, and DENV4, which were first discovered during the 1950s. Currently, they are asymptomatic and have the potential to be life-threatening, displaying a broad range of clinical manifestations [2]. Various manifestations include dengue fever (DF), dengue hemorrhagic fever (DHF), and dengue shock syndrome (DSS). Dengue fever causes significant illness and has widespread morbidity and mortality [4].

A diagnosis is confirmed by isolating the virus from the blood during the viremia phase or by detecting dengue IgM and IgG antibodies using capture-ELISA (enzyme-linked immunosorbent assay) during the post-viremia phase. Virus isolation is infrequently used in the diagnosis of dengue infection. Dengue virus non-structural protein 1 (NS1) antigen by ELISA is routinely used for diagnosis. The polymerase chain reaction technique is the preferred approach for detecting viral genomes [2].

Macrophage infection plays a crucial role in the development of DF and the progression to DHF or DSS. Once a person is infected with a specific serotype, they develop immunity exclusively to that particular serotype for life, not to other serotypes. Infection with a different serotype might lead to severe consequences due to the development of ineffective antiviral antibodies. Sub-neutralizing levels therefore increase the virus's infectivity. Activation of complement and the release of cytokines and other mediators might lead to inflammation-induced capillary leakage, potentially resulting in hemorrhagic symptoms, as observed in our case. Blood volume depletion results in shock. Disseminated intravascular coagulation may occur in patients with severe cases [4].

The pathophysiology of neurological manifestations by dengue infection remains unclear to date, but that could be because of the neurotropic nature of the virus and the immune-mediated injury. The condition affects both the central nervous system and peripheral nervous system during an acute phase, resulting in various neurological complications such as meningitis, encephalitis, encephalopathy, myelitis, mononeuropathy, and polyneuropathy [2]. Involvement of the spinal cord is rare and is seen mostly as transverse myelitis. Our patient has developed transverse myelitis, which is an unusual manifestation among these. Approximately 10 cases have been documented so far. Thereafter, the post-infectious phase might be associated with Guillain-Barré syndrome, oculomotor palsy, neuromyelitis optica, optic neuritis, and disseminated encephalomyelitis [5].

Transverse myelitis often develops over a period of two to 15 days, either as a para-infectious or post-infectious form. The development of neurological symptoms, along with the initial dengue infection (para-infectious) and flaccid paraplegia, are attributed to direct viral invasion of the nervous tissue, whereas the delayed appearance of neurologic symptoms (post-infectious) and spastic paraplegia are considered immunologically mediated neural injury [1]. Direct virus invasion can occur in the para-infectious stage, whereas immune-mediated mechanisms are involved in the post-infectious phase. Post-infectious immune-mediated myelitis develops one to two weeks after the initial symptoms [6].

Evidence of the dengue virus directly invading the central nervous system is found in cases of transverse myelitis, where the virus antigen is isolated from cerebrospinal fluid and spinal cord tissue following dengue infection. A high IgM/IgG ratio in the cerebrospinal fluid is helpful in diagnosing a direct viral infection of the dengue virus [2].

The diagnostic criteria according to the Transverse Myelitis Consortium Working Group 2002 consists of motor, sensory, or autonomic dysfunction, which can be attributed to spinal cord involvement. Signs and symptoms of transverse myelitis typically manifest bilaterally, with the possibility of being asymmetrical or symmetrical. The primary indicator is the presence of a clearly delineated sensory level. Signs of inflammation are shown as pleocytosis in the cerebrospinal fluid analysis or indication of inflammation on the gadolinium-enhanced MRI spine. One must remember that a normal MRI spine does not rule out the clinical diagnosis of transverse spinal cord syndrome [7].

In the treatment of transverse myelitis, a high-dose IV methylprednisolone administration is typically the initial line of treatment leading to faster recovery and an improved neurological outcome. Plasma exchange might be considered as the second choice if not improving with high-dose steroids. The prognosis is variable, and the outcome is uncertain. Physiotherapy and rehabilitation exercises are prescribed for residual neurological deficits and bowel and bladder involvement [6].

## Conclusions

Longitudinally extensive transverse myelitis is a very rare complication associated with dengue fever. It is crucial to promptly identify and observe for neurological complications early. Intravenous immunoglobulins were preferred in our patient. Dengue fever should be included in the differential diagnosis of para-infectious and post-infectious myelitis. We must be conscious of the rare neurological symptoms of dengue fever especially during outbreaks, during and after treatment to facilitate prompt diagnosis and provide appropriate management.
